# Development of magnetic anchoring and guidance systems for minimally invasive surgery

**DOI:** 10.4103/0970-1591.70585

**Published:** 2010

**Authors:** Sara L. Best, Jeffery A. Cadeddu

**Affiliations:** Department of Urology, University of Texas Southwestern Medical Centre, Texas, USA

**Keywords:** Magnetic anchoring and guidance systems, magnets, laparoscopy, natural orifice translumenal endoscopic surgery, laparoendoscopic single site surgery

## Abstract

Recent advances in urology have included natural orifice translumenal endoscopic surgery (NOTES) and laparoendoscopic single-site surgery (LESS). These techniques seek to minimize morbidity by reducing the number of transabdominal port sites, but this comes at a cost of decreased instrument agility and other technical challenges that have prevented LESS and NOTES from entering mainstream urologic practice. Magnetic anchoring and guidance systems (MAGS) consist of instruments that are inserted laparoscopically through an entry in the peritoneal cavity at one point and then driven into position elsewhere and controlled with magnets. These instruments improve the ergonomics of minimally invasive surgery and may help make LESS and NOTES more accessible to urologists across experience levels.

## INTRODUCTION

Since the first report of a laparoscopic nephrectomy by Clayman *et al*,[1] the advantages of laparoscopic surgery in urology over open techniques in many situations have been repeatedly demonstrated. With its evolution, patients undergoing laparoscopic nephrectomy have enjoyed a decreased blood loss, shorter hospital stay, convalescence, less pain, and improved cosmesis.[2,3] Although the complication rate of laparoscopic nephrectomy is equivalent to that of open nephrectomy,[4] it still requires 3-4 transabdominal incisions, each of which carries the risk of port-site bleeding, hernia, or internal organ injury, as well as increased number of scars.[5–7]

Concerns about these morbidities have motivated the search for ways to make laparoscopy even less invasive. Natural orifice translumenal endoscopic surgery (NOTES) and laparoendoscopic single site surgery (LESS) surgery are designed to have fewer, if any, transabdominal port sites and may thereby help mitigate some of these risks. In NOTES, operative instruments are passed through an opening created in a natural orifice, such as the stomach, colon, or vagina, leaving the patient with no visible scars. Similarly, LESS surgery involves passing multiple surgical instruments through a solitary transabdominal incision, often at the umbilicus, where the scar can be hidden.

**Figure 1:**
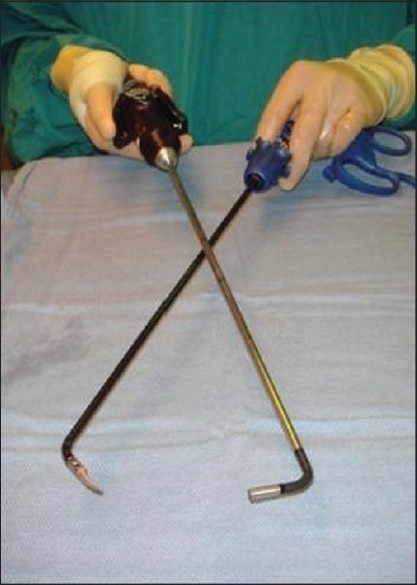
Articulating laparoscopic camera and grasper. The instruments are being held to simulate how they are used in LESS surgery, with the instruments crossed proximally.

However, this potential decrease in morbidity comes with additional challenges. One of the principal tenets of laparoscopy is the triangulation of instruments, whereby ports are spaced out across the abdominal wall to allow each instrument to approach the operative site from a different trajectory. This spacing of port sites facilitates the grasping and manipulation of tissues. In LESS and NOTES, however, all of the instruments are typically passed into the abdomen through a solitary insertion site. This difference can lead to instrument collision or “swordfighting” as both dissecting instruments and the camera compete for the same working space. Some of these difficulties have been improved by the development of articulating instruments that “bow out” away from the trajectory of insertion, simulating triangulation [Figure 1]. These instruments also cross at a proximal fulcrum, outside the field of view, to further reduce collisions. Although these advances have made even complex LESS and NOTES urologic procedures feasible in experienced hands,[8–10] these instruments require mastery of an additional skill set. For example, crossed articulating instruments mandate that the surgeon adapt to the fact that the instrument that appears on the left side of the screen is actually being controlled by the right hand and vice versa. Similarly, use of a 5-mm flexible laparoscope can help diminish instrument collision and improve the viewing angles, but this comes at the cost of decreased optical quality and a potentially more-challenging job for the assistant surgeon.[11,12]

**Figure 2:**
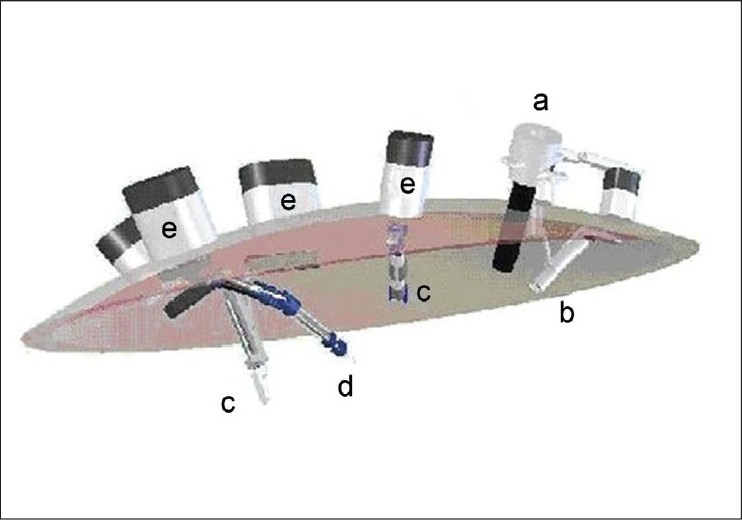
Schematic representation of MAGS platform. One conventional trocar is depicted with 4 deployed MAGS instruments. (a) deployment trocar; (b) MAGS camera; (c) retractors; (e) robotic cauterizer; and (e) external magnets.[31]

Although these challenges are surmountable to skilled laparoscopists, LESS and NOTES will be more likely to take off in mainstream practice if these techniques are made easier with further instrument development. One way to accomplish this would be through the creation of “fully insertable” instruments that do not take up port site space during the operation. To this end, researchers in several surgical fields, including urology, are developing instruments that harness magnetic forces to steer and operate completely insertable intracorporeal instruments via externally controlled magnets. Such technology, called magnetic anchoring and guidance systems (MAGS), includes cameras, retractors, dissectors, cautery devices, and even combinations thereof [Figure 2]. In this article, we describe the development and current status of these instruments.

## MAGNETIC ANCHORING AND GUIDANCE SYSTEMS DEVELOPMENT

MAGS instruments typically consist of 2 parts: an external handheld magnet and an insertable magnetic intracorporeal device, such as a camera, retractor, and so on [Figure 2]. The internal component is inserted through the pre-existing incision (usually the LESS or NOTES point of peritoneal access) and is then coupled via magnetic attraction across the body wall to the external component. By moving the external handheld magnet around the patient’s abdominal wall, the internal device can be steered to the task-appropriate location. Its position can then be adjusted as needed, such as to alter the view if using a MAGS camera, to lift the liver edge in the case of a MAGS retractor, or even continuously as when using a MAGS cautery device.

Instruments of this type were first reported by Park *et al*.[13] in 2007, when an insertable MAGS camera was used in combination with an LED-wrapped trocar that functioned as a light source. By using this camera for visualization along with MAGS retractors, the authors were able to perform 2 porcine nephrectomies, using only 2 standard laparoscopic ports. After the development of a MAGS cautery device later that year, the same group reported performing 2 LESS porcine nephrectomies, using only a 15-mm transabdominal trocar along with their MAGS camera, cautery, and retractors.[14] Some of the difficulties they found in these investigations were substandard image quality related to the relatively low resolution of the camera modified for use in this project along with lens fogging because the camera cannot be removed, cleaned, and reinserted as easily as a standard laparoscopic camera. Also, relative weakness of the magnets meant that the devices could only be reliably coupled across a 1.5-cm thick porcine abdominal wall before the intracorporeal component would “decouple” or fall off the anterior abdominal wall.[13,14]

## FURTHER ADVANCES IN MAGS AND MAGNETIC PLATFORMS

**Figure 3:**
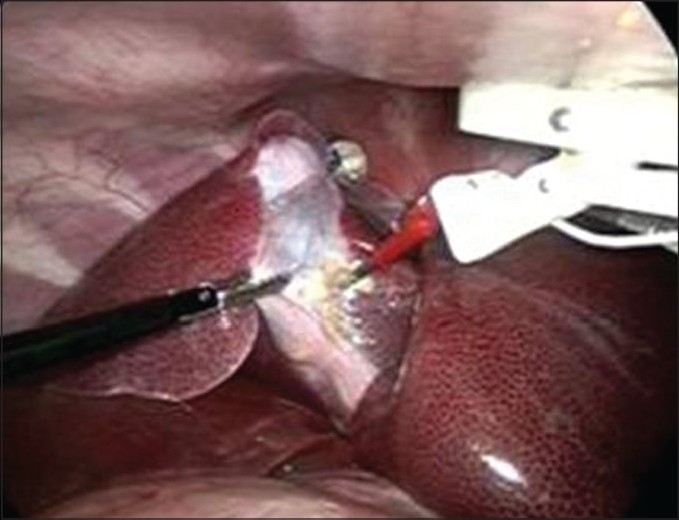
MAGS pneumatically controlled cautery arm (right) being used to perform a porcine cholecystectomy. The device is being suspended from the anterior abdominal wall by an external magnet, held by the surgeon

Given the technical challenges this group encountered, a multidepartmental consortium at the University of Texas Southwestern Medical Center was formed to make further improvements. They developed instruments with stronger magnetic coupling as well as a pneumatically activated movable cautery “arm” [Figure 3]. These instruments were used to perform 4 transvaginal NOTES porcine cholecystectomies. The first generation of this new cautery device was limited by the cautery cord being too short to allow adequate mobility in the abdomen. A second generation device with a 40-cm cautery cord did allow for successful cholecystectomy in 2 nonsurvival animals. A rectal injury occurred in one of these animals, however, and was felt to be related to the removal of the magnetic gall bladder retractors they used during the procedure. The authors also engineered a system whereby the external magnet can be removed and replaced with an 18-gauge needle anchor, which helps avoid the inadvertent “clumping” of magnetic devices if more than one MAGS instrument set is used.[15]

However, this is not the only group investigating magnetic instruments. In response to the difficulties in optimizing visualization of the operative field encountered during LESS surgery using a standard transabdominal endoscope, Fakhry *et al*. designed a wireless, insertable magnetic camera that they tested against a standard 30° laparoscope in a laparoscopic trainer model. The participants in the study were able to find more targets (74.8 compared with 54.7) in a maze created inside the trainer with their magnetic camera than with the standard endoscope, although the magnetic trial took longer (34.9 vs 24.1 min).[16] Of note, although Fakhry and colleagues have not yet reported the use of this device in an animal model, they did report the same experience of periodic “decoupling” of the internal and external components that Zeltser *et al*. noted in their porcine studies.[14,16]

**Figure 4:**
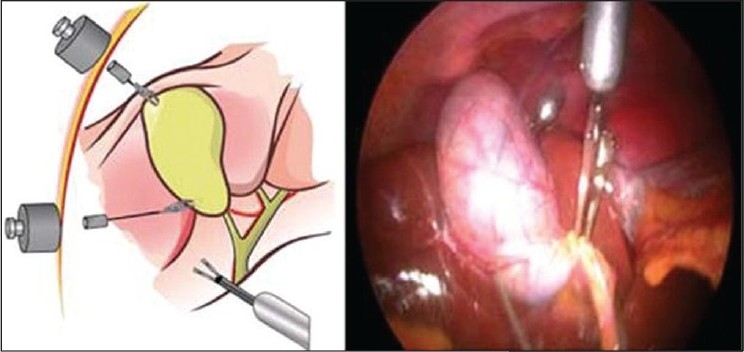
Images from Dominguez *et al*’s article describing their “neodymium magnetic forceps.” Schematic shows external magnets retracting insertable magnetic graspers clipped onto the gallbladder. Photo shows these tools *in vivo*.[18]

Several authors have noted the potential of magnetic retraction devices. One of the challenges of LESS and NOTES surgery, with their limited triangulation, is that it is difficult to provide adequate tissue retraction. Retraction with traditional transabdominal graspers or even articulating ones can impair the view of the operative field as well as conflict with the other operating instruments when they are all passed along the same trajectory through a single incision. Thus, the development of insertable MAGS retractors, which can be deployed into position in the abdomen and manipulated to create suitable exposure without taking up valuable port space, is appealing. Ryou and Thompson[17] reported their development of MAGS instruments that they used to perform NOTES cholecystectomy or simulated ventral hernia repair in a porcine model. Their device consists of a large (4 × 2 × 2 inch) external magnet fixed to a moveable arm, which magnetically attracts several magnetically conjugated “clips” or graspers. These small clips were inserted into the abdomen and attached to either the liver edge or onto a piece of synthetic mesh, depending on the procedure. The magnetic attraction was used to elevate the liver edge in the case of cholecystectomy or to maneuver the mesh and hold it in place during laparoscopic fixation. They found this system to provide adequate elevation of the liver even when the gall bladder was being pulled upon during cholecystectomy and that the magnetic clips greatly eased the location and fixation of the mesh during ventral hernia repair. Similarly, Dominguez *et al*.[18] have described using what they call “neodymium magnetic forceps” to perform 40 human LESS cholecystectomies. These instruments consist of an internal magnet attached to a flexible alligator grasper head. This is then passed intracorporeally and used to grasp and elevate the gall bladder via coupling with a large external magnet [Figure 4]. They report no magnet-related complications or conversions in these 40 patients.

Lehman *et al*.[19] are seeking to combine multiple instruments, including a grasper, cauterizer, and camera into one magnetic “robot,” which can be inserted into the peritoneal cavity and then suspended from the anterior abdominal wall via magnets. The central portion contains the imager and off this extend 2 articulating arms, which are controlled from a separate console with a joystick. They have inserted this robot through a NOTES gastrotomy in 3 nonsurvival pigs and used it to perform various tasks, including abdominal exploration, bowel manipulation, and attempted cholecystectomy. The authors do note that this device was a prototype, and as such, they did encounter some technical difficulties, including coupling failure and mechanical failure of the arm movements. Despite this, the notion of a fully contained, multifunctional magnetically anchored device is intriguing.

## THE FUTURE OF MAGS IN UROLOGY

The use of NOTES and even more so, LESS surgery, are becoming more prominent in urology. Several academic centers have reported series of LESS nephrectomy patients with good outcomes.[20–24] Yet these surgeons will be the first to admit to the challenges of less-invasive techniques, such as NOTES and LESS. As Stolzenburg *et al*.[25] put it, “unusual and inappropriate view angles are observed during LESS,” plus the articulating instrumentation has its own learning curve to overcome. For LESS and NOTES to take off in mainstream urologic practices, technology will need to improve and overcome these challenges.

**Figure 5:**
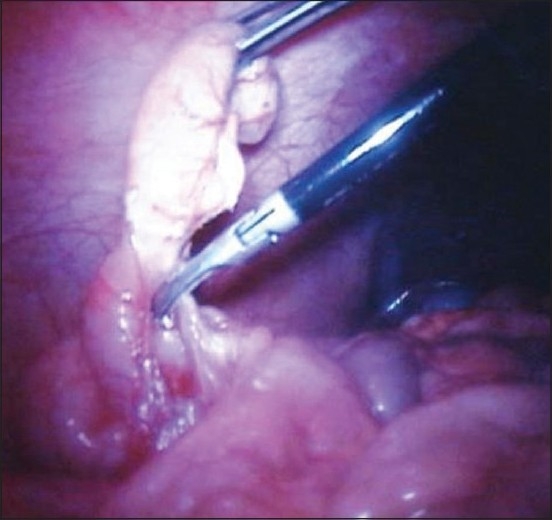
Image provided by MAGS camera during human LESS appendectomy reported by Cadeddu *et al*. This picture shows appendix being elevated, while the appendiceal mesentery is being transected with a ultrasonic shears.[26]

Fortunately, as described, some of the existing prototypes are already showing promise in LESS clinical models. A MAGS camera may be one of the most useful instruments as it will free up the viewing angle from a fixed position at the transabdominal port site and allow the surgeon to move the camera to essentially any point in the abdomen. A camera that can be inserted and moved to a position that recreates the view of traditional multiport laparoscopy may help diminish the learning curve of LESS and NOTES. Cadeddu *et al*.[26] have already reported using a MAGS camera to perform a LESS nephrectomy on a 50-year-old woman with a nonfunctioning kidney, as well as a LESS appendectomy in a 12-year-old boy [Figure 5]. They described the image quality as comparable to a traditional 5-mm laparoscope and found that they experienced much less “swordfighting” of the operating instruments with the MAGS camera because there was no laparoscope competing for space in the LESS port. The authors did not report any imprinting of the skin from the magnets nor any loss of pneumoperitoneum despite the passage of the camera wires next to the transabdominal port across the abdominal wall. The camera lens, which cannot be easily removed to be cleaned if it fogs, was cleaned twice during the nephrectomy with irrigation and a small gauze swab. They were careful to point out, however, that both patients in this report were relatively thin, with abdominal walls measuring 2.5 and 1.2 cm thick, respectively, although these instruments have been shown to stay coupled at up to 4.3 cm drop-off distance in an ex vivo model. Because magnetic attraction decreases exponentially with greater distance, the thicker abdominal walls encountered in the obese may present a problem. Further research will need to be done to both assess the distance at which available instrument prototypes can be used and also to develop ones that can work in overweight patients.

## CONCLUSION

LESS has been reported in the literature for appendectomy and cholecystectomy since the 1990s,[27–30] but this technique did not “take off” until the development of more facile instrumentation in the last few years. It has been even slower to take hold in urology, where the larger working envelopes and complex vasculature of the retroperitoneum have made instrumentation even more critical. We find the MAGS technology ideally situated to help further ameliorate the challenges of LESS and NOTES through re-creation of the greater triangulation and more comfortable viewing angles experienced with traditional multiport laparoscopy. Further development of these tools will probably help single-incision surgery enter the mainstream of urology.
